# The Heresy of Hypoabsorptive Bariatric Surgery: A Critical Reappraisal of Long-Term Outcomes and Clinical Trade-Offs

**DOI:** 10.1007/s11695-026-08749-4

**Published:** 2026-05-28

**Authors:** Francesco Saverio Papadia

**Affiliations:** 1 University of Genoa, Department of Surgical Sciences and Integrated Diagnostics, Genoa, Italy; 2 Ospedale Policlinico San Martino, Genoa, Italy

## Abstract

Hypoabsorptive bariatric procedures remain underutilized despite offering superior durable weight loss and metabolic comorbidity resolution. This review examines evidence from registry analyses, comparative studies, and long-term cohorts with follow-up extending to 30 years. When adjusted for baseline characteristics, hypoabsorptive procedures do not incur higher 30-day morbidity than RYGB. Five-year data confirm superiority in total weight loss (40.6% versus 33.8% for RYGB) and diabetes remission (92.8%). However, long-term follow-up reveals substantial nutritional costs affecting > 80% of patients in cohorts that often include patients treated with historical techniques, with cumulative reoperation rates reaching 37% at 10 years. Complication timing correlates with patient frailty—older age, lower BMI, and greater comorbidity burden predict earlier decompensation. Hypoabsorptive surgery offers unmatched efficacy at substantial long-term cost that manifests earlier in vulnerable populations, demanding rigorous patient selection and honest acknowledgment of trade-offs.

## Key Points

Hypoabsorptive procedures achieve 40.6% total weight loss at 5 years, sustained for decades.

In long-term cohorts, which often include patients treated with historical techniques, nutritional complications affect > 80% of patients and accumulate over decades. Modern limb length optimization has reduced the risk of severe protein malnutrition, but micronutrient deficiencies remain a substantial challenge.

Reoperation rates reach 37% at mean 4.4 years, with revisions needed as late as 29 years postoperatively.

Frailer patients (older, lower BMI, more comorbidities) decompensate earlier and more severely.

## Introduction

*“Heresy: dissent or deviation from a dominant theory*,* opinion*,* or practice.”*

This definition captures the position of hypoabsorptive bariatric surgery in contemporary practice. Surgeons who perform these operations find themselves defending a position that contradicts generally accepted beliefs, facing skepticism from colleagues who view these procedures as carrying unacceptable risks [1, 2].

Despite offering superior and durable weight loss along with exceptional metabolic comorbidity resolution, hypoabsorptive procedures constitute less than 3% of bariatric operations in the United States [3]. An international survey identified primary barriers to adoption: perceived high long-term complication rates (43.5%), lack of training (38.1%), and low patient demand (31.5%) [2]. Yet contemporary evidence challenges these assumptions, demonstrating that when adjusted for baseline characteristics, hypoabsorptive procedures carry short-term risk profiles comparable to established operations [4].

This narrative review critically examines the evidence base for hypoabsorptive bariatric surgery, weighing its remarkable long-term efficacy against accumulating clinical costs that become fully apparent only decades after operation. Particular attention is paid to the observation that complications occur earlier in frailer patients—those with older age, lower nutritional reserves, and greater comorbidity burden—and that Publio Terenzio Afro’s observation, “*Senectus ipse est morbus*” (old age itself is disease), proves tragically apt.

## Methods

This narrative review was conducted following a structured search of the PubMed and Scopus databases for articles published between January 2000 and December 2023. The primary search strategy employed combinations of the following keywords: “hypoabsorptive bariatric surgery,” “biliopancreatic diversion,” “duodenal switch,” “SADI-S,” “single anastomosis duodeno-ileal bypass,” “long-term outcomes,” “nutritional complications,” “reoperation,” and “frailty.” We included original research articles, systematic reviews, meta-analyses, and large registry studies (e.g., MBSAQIP) that reported on outcomes of hypoabsorptive procedures. Priority was given to studies with a minimum follow-up of 5 years and those comparing hypoabsorptive techniques to Roux-en-Y gastric bypass. References were also manually screened to identify additional relevant studies.

## Hypoabsorptive Surgery: Current State

According to the ASMBS 2022 estimate, hypoabsorptive procedures constitute a minority of bariatric operations in the US [3]. Sleeve gastrectomy remains dominant at 57.4%, while RYGB accounts for 22.2%. BPD/DS procedures represent 2.2% of cases, with SADI-S and OAGB accounting for 0.6% and 0.4% respectively. The IFSO 8th Global Registry Report (2024) confirms this pattern globally [5].

## Defining Hypoabsorption

Hypoabsorption entails intestinal adaptation phenomena that increase immediate postoperative digestive absorption capacity—the residual hypoabsorption represents a key mechanism of long-term weight maintenance [6]. This adaptation process explains both durability of weight loss and potential for late nutritional complications. Critically, very long-term studies show adaptation does not eliminate complications; it merely delays clinical manifestation [7, 8].

## Perioperative Safety

### Leak Rates and Predictors

Analysis of the MBSAQIP database identified 177 leaks (0.8%) among 21,839 DS patients within 30 postoperative days, approximately double the 0.4% rate for RYGB [9]. Significant predictors included steroid use (aOR = 3.01), age (each decade: 26% increased risk), and operative length. Leak rates decreased over time from 1.8% in 2015 to 0.4% in 2022 [9].

### Propensity-Matched Comparisons with RYGB

Propensity-matched analysis comparing 30-day outcomes between BPD/DS and RYGB (5,050 patients per cohort) found surgical site infections higher in RYGB (1% vs. 0.5%, *p* = 0.007) and blood transfusions higher in RYGB (1.1% vs. 0.6%, *p* = 0.018), with no differences in mortality, readmission, or reoperation [4]. When matched for baseline characteristics, BPD/DS does not incur higher 30-day morbidity than RYGB.

### SADI-S Versus OAGB

Comparative analysis of SADI-S and OAGB demonstrated higher grade 2 complications (2.6% vs. 0.8%) and readmission (3.7% vs. 1.9%) after SADI-S, reflecting patient selection of higher-risk patients [10]. Absolute complication rates remained low for both procedures.

### Long-Term Efficacy

#### Weight Loss Outcomes

MBSAQIP data demonstrated %TWL at 24 months highest in DS (40.6%) versus RYGB (33.8%) and sleeve gastrectomy (28.5%) [3]. Sethi et al. reported sustained %EWL of 65.1% at 2 years, 63.8% at 5 years, and 67.9% at 10–15 years after BPD/DS [11]. Pujol Gebelli et al. found DS superior to SADI-S at 5 years: %TWL 42.1% vs. 36.0% [12].

The longest follow-up data—30 years after BPD—demonstrate remarkable durability: mean %TWL was 32.8% at 1 year and 37.7% at 30 years [8]. This challenges any intervention to match hypoabsorptive durability.

#### Metabolic Comorbidity Resolution

Diabetes remission after hypoabsorptive procedures is consistently high: 92.8% for DS and 85.7% for SADI-S [12]. Hypertension remission was 95.2% for DS versus 85.1% for SADI-S. In patients with type 2 diabetes and severe obesity undergoing BPD, fasting glucose normalizes in > 90%, with recurrence only occasional [7, 13].

In overweight and class I obese patients with diabetes (BMI 25–35 kg/m²), 48% maintained diabetes remission at 10 years, but at substantially higher complication cost [14].

Koh et al. demonstrated revisional DS achieved 100% diabetes improvement and 69% remission, confirming hypoabsorptive surgery as an effective revisional strategy [15].

#### The Frailty Hypothesis: Complication Timing and Patient Vulnerability

A critical pattern emerges from long-term studies: complications concentrate earlier and more severely in frailer patients. Three studies demonstrate this “frailty gradient,” which is summarized in Table 1.

**Table 1:** The frailty gradient: patient characteristics and complication timing after hypoabsorptive procedures

Cohort Description	Mean Age (years)	Mean BMI (kg/m²)	Key Comorbidities	Complication Onset	Severe Complication Rate	Reoperation Rate	Mortality	Source
Patients with severe obesity	38	48.7	46% with T2DM, 62% with HTN	Progressive over 30 yrs; 6%→74%	74% at 30 yrs	6% at mean 10 yrs	12% at 30 yrs	[8]
Patients with severe obesity and T2DM	52	49.7	100% with T2DM, 96% with HTN	Accelerated; 50% at 10–20 yrs	50% at 10–20 yrs	11%	27% at 10–20 yrs	[7]
Patients with overweight/Class I obesity and T2DM	57	30.6	100% with T2DM, 53% with HTN	Early and severe; 53% within 10 yrs	53% within 10 yrs	33% within 10 yrs	10% within 10 yrs	[14]

T2DM = type 2 diabetes mellitus; HTN = hypertension; BMI = body mass index; yr = year

#### The 30-Year BPD Cohort

In 199 patients followed 30 years post-BPD, nutritional complications accumulated progressively: 6% at 1 year, 22% at 5 years, 29% at 10 years, 60% at 20 years, and 74% at 30 years. Revisional surgery was required as late as 29 years postoperatively, with mean age at revision of 55 years. Surgical complications concentrated early; nutritional complications increased progressively [8]. 

#### Patients with Type 2 Diabetes and Severe Obesity

In 173 patients with diabetes followed 10–20 years after BPD, despite 92% maintaining normal fasting glucose, severe complications affected nearly 50%. Mortality was 27% in operated patients versus 13% in matched controls (*p* < 0.02) [7]. This finding is highly controversial and challenges the long-held assumption that the metabolic benefits of bariatric surgery uniformly translate into a net survival advantage. The authors of the original study postulated that the negative effects of BPD tend to worsen over time, with no evidence of intestinal adaptation sufficient to prevent late malnutrition, metabolic bone disease, and other complications. They suggested that the cumulative burden of these sequelae may offset the survival benefits typically associated with diabetes remission. While residual confounding cannot be excluded, given that the decision to operate was not randomized, the magnitude of the mortality difference warrants serious consideration. Clinically, this finding underscores that in hypoabsorptive procedures, the long-term trade-off is not merely one of quality of life, but potentially of survival itself in a subset of vulnerable patients. It reinforces the need for rigorous patient selection and lifelong surveillance, and it argues against the use of such procedures in patients with limited nutritional reserve or reduced ability to tolerate chronic malabsorption.

#### Overweight/Class I Obese Patients with Diabetes

In the frailest population—30 patients with BMI 25–35 kg/m²—severe complications developed in 53% within 10 years, with 33% requiring surgical revision and 10% mortality, despite only 48% achieving diabetes remission. Complication occurrence was unrelated to diabetes remission status [14]. 

#### Synthesis: The Frailty Gradient

Table 1 summarizes the relationship between patient characteristics and complication timing. Frailer patients (older, lower BMI, greater comorbidity) decompensate earlier and more severely.

#### Why Frailer Patients Decompensate Earlier

Mechanisms include: reduced nutritional reserves (lower BMI patients lack nutritional redundance), age-related physiological changes reducing adaptive capacity, comorbidity burden impairing nutritional homeostasis, and dietary limitations (vegetarians at higher risk for protein malnutrition) [11, 14, 16].

### Long-Term Nutritional and Surgical Complications

#### Nutritional Deficiencies

In studies reporting on very long-term follow-up, often including patients who underwent historic configurations with shorter common channels, the prevalence of nutritional deficiencies is substantial. As detailed in Table 2, Sethi et al. documented prevalence after BPD/DS: vitamin D 89%, vitamin K 65%, zinc 65%, anemia 57%, protein deficiency 40% [11]. In the 30-year cohort, metabolic bone disease affected 45% and micronutrient deficiency 42% at 30 years [8].

**Table 2:** Prevalence of nutritional deficiencies in patients after hypoabsorptive procedures

Deficiency	Prevalence (%)	Time Point	Patient Population	Source
Vitamin D	89	Mean 8.2 yrs	Patients after BPD/BPD/DS	[11]
Vitamin K	65	Mean 8.2 yrs	Patients after BPD/BPD/DS	[11]
Zinc	65	Mean 8.2 yrs	Patients after BPD/BPD/DS	[11]
Anemia	57	Mean 8.2 yrs	Patients after BPD/BPD/DS	[11]
Protein deficiency	40	Mean 8.2 yrs	Patients after BPD/BPD/DS	[11]
Metabolic bone disease	45	30 years	Patients after BPD	[8]
Micronutrient deficiency	42	30 years	Patients after BPD	[8]

BPD = biliopancreatic diversion; DS = duodenal switch

Kellum et al. reported distal RYGB with 50-cm common channel required revision in 56.5% for protein malnutrition versus 32% with ≥ 100-cm channel [17]. Jammu and Sharma found hypoalbuminemia in 13.1% of MGB patients with bypass > 250 cm, becoming rare when standardized to 200 cm [16].

#### Surgical Complications and Reoperation Rates

Table 3 summarizes surgical complications requiring reoperation. Sethi et al. reported 37% developed complications requiring surgery at mean 4.4 years: incisional hernia 9%, internal hernia 8%, suboptimal weight loss requiring revision 8%, small bowel obstruction 7%, severe malnutrition 4% [11]. At 10 + years, reoperation rate reached 41%.

**Table 3:** Surgical complications requiring reoperation in patients after hypoabsorptive procedures

Complication	Rate (%)	Time Point	Patient Population	Source
Incisional hernia	9	Mean 4.4 yrs	Patients after BPD/BPD/DS	[11]
Internal hernia	8	Mean 4.4 yrs	Patients after BPD/BPD/DS	[11]
Suboptimal weight loss requiring revision	8	Mean 4.4 yrs	Patients after BPD/BPD/DS	[11]
Small bowel obstruction	7	Mean 4.4 yrs	Patients after BPD/BPD/DS	[11]
Severe malnutrition requiring revision	4	Mean 4.4 yrs	Patients after BPD/BPD/DS	[11]
Cumulative reoperation rate	37	Mean 4.4 yrs	Patients after BPD/BPD/DS	[11]
Cumulative reoperation rate	41	10 + yrs	Patients after BPD/BPD/DS	[11]
Distal RYGB revision (50-cm common channel)	56.5	20–25 yrs	Patients after distal RYGB	[17]
Distal RYGB revision (≥ 100-cm common channel)	32	20–25 yrs	Patients after distal RYGB	[17]

BPD = biliopancreatic diversion; DS = duodenal switch; RYGB = Roux-en-Y gastric bypass

In the 30-year cohort, 6% required revision at mean 10 years (range 1–29) [8].

#### Mechanistic Insights

Li et al. demonstrated BPD/DS produces intestinal hypertrophy and elevated GLP-1/PYY, effects not seen after sleeve gastrectomy alone [18]. Werling et al. found enhanced energy expenditure after BPD/DS related to favorable body composition [19]. Pucci and Batterham detailed gut hormone changes sustained up to 10 years postoperatively [20].

The critical insight: intestinal adaptation does not eliminate malabsorption—it establishes a new equilibrium vulnerable to disruption by aging, intercurrent illness, or inadequate intake [7, 8].

#### The Illusion of “Lifelong Follow-up”

When advocating lifelong follow-up, we must acknowledge this is unlikely universally achievable. The 30-year cohort experienced substantial attrition, with only 19% remaining in follow-up at 30 years [8].

Even with optimal follow-up and modern surgical techniques (e.g., longer common channels, routine supplementation protocols), some nutritional complications may be unavoidable, particularly in vulnerable patients. In cohorts treated with historic configurations, nutritional complications appeared to accumulate relentlessly; however, contemporary limb length optimization has likely attenuated the most severe forms of protein malnutrition, though micronutrient deficiencies remain a persistent challenge [8].

#### Patient Selection and Shared Decision-Making

Factors associated with favorable outcomes: lower preoperative BMI (< 50), younger age, longer common channel (≥ 100 cm), shorter bypass limb (≤ 200 cm), compliance with follow-up. Factors associated with complications: steroid use, advanced age, vegetarian diet, diabetic nephropathy, liver disease [4, 9, 14, 16].

The overweight/class I obese patient with diabetes represents a particularly high-risk population and should generally not be offered primary hypoabsorptive procedures [14].

#### Optimizing Hypoabsorptive Surgery

##### Limb Length Optimization

Current practice favors DS with 200-cm alimentary limb and 100-cm common channel [12], SADI-S with 300-cm common channel [12], and MGB with 200-cm bypass [16]. The historic 50-cm common channel is associated with unacceptable late complication rates [17], and should be considered obsolete in contemporary practice.

##### Staged Approaches

Initial sleeve followed by conversion allows assessment of compliance before committing to hypoabsorptive reconstruction.

##### Enhanced Perioperative Care

Steroid minimization, preoperative nutritional optimization, experienced surgical teams, routine mesenteric defect closure, structured follow-up.

##### Adjuvant Pharmacotherapy

GLP-1 agonists may manage weight recurrence, potentially reducing need for revisional surgery.

## Discussion

Hypoabsorptive procedures offer unmatched efficacy—sustained weight loss > 30–40% TWL, diabetes remission > 90%, cardiovascular risk reduction persisting for decades. Yet these benefits are purchased at substantial cost that varies systematically with patient characteristics.

Three lines of evidence support a “frailty gradient”:

1. The 30-year cohort: nutritional complications rise from 6% at 1 year to 74% at 30 years [8].
2. Diabetes patients: complication rates > 50% at 10–20 years with higher mortality than controls [7].
3. Overweight diabetic patients: complication rates > 50% within 10 years, 33% revision, 10% mortality [14].

Even with meticulous follow-up and modern surgical techniques that employ safer limb lengths (e.g., ≥ 100 cm common channel, ≤ 200 cm bypass limb), the risk of late nutritional complications remains significant, particularly in vulnerable populations. However, it is important to distinguish between historical procedures, which used ultra-short common channels (e.g., 50 cm) and were associated with substantially higher rates of protein malnutrition requiring revisional surgery, and contemporary optimized techniques, where severe protein malnutrition requiring intervention occurs in 4–14% of patients. The burden of micronutrient deficiencies, while reduced, has not been eliminated. The data suggest that while modern optimization reduces the risk of the most severe forms of protein malnutrition, it does not eliminate the chronic, cumulative burden of micronutrient deficiencies. The concept of “lifelong follow-up” is necessary but insufficient; attrition is inevitable and the long-term burden of complications remains a central consideration in patient selection.

## Conclusions

Hypoabsorptive bariatric surgery is remarkably effective. Weight loss > 30–40% TWL sustained for decades, diabetes remission > 90%, cardiovascular risk reduction superior to any other intervention.

Short-term safety profiles are acceptable in experienced hands. Propensity-matched analyses demonstrate no higher 30-day morbidity than RYGB when adjusted for baseline characteristics.

Long-term nutritional complications are substantial, cumulative, and follow a predictable frailty gradient. Vitamin D deficiency affects nearly 90% in long-term cohorts (many of which include historical techniques), protein malnutrition requiring intervention in 4–14% with modern limb lengths, and reoperation rates 37–41% at 10 years. While modern limb length optimization reduces the risk of severe protein malnutrition, the burden of micronutrient deficiencies remains significant. Complications manifest earlier and more severely in frailer patients, those with lower BMI, advanced age, greater comorbidity burden.

Patient selection is paramount. Ideal candidates are those willing and able to commit to lifelong surveillance, with adequate nutritional reserves. The overweight/class I obese diabetic patient represents a particularly high-risk population.

Limb length optimization improves safety. Standardization to ≥ 100 cm common channel reduces malnutrition risk while preserving efficacy.

“Lifelong follow-up” is necessary but insufficient. Even with meticulous surveillance, the long-term burden of nutritional deficiencies remains substantial, and attrition from follow-up is inevitable.

The heresy of hypoabsorptive surgery lies not in performing these operations, but in advocating them without confronting their uncomfortable truth: chronic follow-up is unlikely universally achievable, and the full magnitude of complications may become evident only in the very long term. As Publio Terenzio Afro observed 160 b.c., “*Senectus ipse est morbus*”—old age itself is disease.

For appropriately selected patients willing to commit to lifelong surveillance, hypoabsorptive surgery delivers benefits that no other intervention can match. The heresy, perhaps, is not in performing these operations, but in failing to offer them to those who might benefit most while fully informed of the substantial trade-offs involved.

## Data Availability

No datasets were generated or analysed during the current study.
